# Bufadienolides from Chansu Injection Synergistically Enhances the Antitumor Effect of Erlotinib by Inhibiting the KRAS Pathway in Pancreatic Cancer

**DOI:** 10.3390/ph17121696

**Published:** 2024-12-16

**Authors:** Yanli Guo, Yu Jin, Jie Gao, Ding Wang, Yanming Wang, Liya Shan, Mengyu Yang, Xinzhi Li, Ketao Ma

**Affiliations:** 1 Key Laboratory of Xinjiang Endemic and Ethnic Diseases, Ministry of Education, Shihezi University School of Medicine, Shihezi 832003, China; guo1040927699@126.com (Y.G.); 15090332205@139.com (Y.J.); zerowangding@163.com (D.W.); wangyanminglyo@163.com (Y.W.); shanliya0126@shzu.edu.cn (L.S.); 18290749397@163.com (M.Y.); 2 NHC Key Laboratory of Prevention and Treatment of Central Asia High Incidence Diseases, First Affiliated Hospital, Shihezi University School of Medicine, Shihezi 832003, China; 3 Department of Physiology, Shihezi University School of Medicine, Shihezi 832003, China; 4 Institute of Physiology, School of Basic Medical Sciences, Lanzhou University, Lanzhou 730000, China; gaoj2023@lzu.edu.cn; 5 Department of Pathophysiology, Shihezi University School of Medicine, Shihezi 832003, China

**Keywords:** Chansu injection, bufadienolides, *Venenum Bufonis*, erlotinib, pancreatic cancer, KRAS

## Abstract

**Background and Objectives:** The Chansu injection (CSI), a sterile aqueous solution derived from Chansu, is applied in clinical settings to support antitumor and anti-radiation treatments. CSI’s principal active components, bufadienolides (≥90%), demonstrate potential effects on pancreatic cancer (PDAC), but their underlying mechanisms remain unclear. This study aimed to elucidate the antitumor effects and pathways associated with CSI in PDAC. **Methods:** Network pharmacology and bioinformatics analyses explored CSI’s mechanisms against PDAC. MTT, colony-formation, and migration assays evaluated CSI’s impact on proliferation and migration in PANC-1 and MIA PACA-2 cells, both as a single agent and in combination with erlotinib (EGFR inhibitor). Cell cycle analysis employed flow cytometry. Animal experiments were performed on tumor-bearing mice, with targets and pathways assessed via molecular docking and western blotting. **Results:** CSI treatment suppressed PDAC cell proliferation and migration by inducing G2/M phase arrest. Network pharmacology, bioinformatics, and molecular docking indicated that CSI’s anti-PDAC effects may involve EGFR pathway modulation, with CSI lowering p-EGFR/KRAS/p-ERK1/2 pathway expressions in PDAC cells. Additionally, sustained KRAS activation in mediating erlotinib resistance in PDAC and CSI potentiated erlotinib’s antitumor effects through enhanced KRAS and p-ERK1/2 inhibition. CSI also enhanced erlotinib’s efficacy in tumor-bearing mice without causing detectable toxicity in renal, cardiac, or hepatic tissues at therapeutic doses. **Conclusions:** CSI as an adjuvant used in antitumor and anti-radiation therapies enhanced erlotinib’s antitumor effects through modulation of the KRAS pathway. CSI and erlotinib’s synergistic interaction represents a promising approach for addressing erlotinib resistance in PDAC treatment.

## 1. Introduction

Projected to rank as the second-leading cause of cancer mortality by 2030, pancreatic ductal adenocarcinoma (PDAC) remains one of the most deadly malignancies [1]. Currently, only 20% of PDAC cases are eligible for surgical resection, the principal treatment option, while the majority—often diagnosed at advanced or metastatic stages—lack access to potentially curative intervention [2]. Although a range of targeted drug therapies has been explored, these efforts have yet to achieve substantial improvements in survival outcomes [3].

The epidermal growth factor receptor (EGFR) family is integral to the growth and development of normal tissues but is frequently overexpressed in pancreatic cancer, where the enhanced autophosphorylation of EGFR drives key oncogenic pathways, such as RAS Proto-Oncogene, GTPase (RAS) -Raf1 Proto-Oncogene (RAF)- mitogen-activated protein kinase (MAPK), and phosphatidylinositol 3-kinase (PI3K)/protein kinase B (AKT). These pathways support sustained cellular proliferation, angiogenesis, and metastasis in pancreatic cancer [4,5]. Therapeutic interventions, including EGFR inhibitors and monoclonal antibodies like erlotinib, gefitinib, cetuximab, and bevacizumab, aim to manage disease progression. Nonetheless, resistance or insensitivity to these treatments often emerges rapidly, posing a formidable obstacle to improving patient outcomes and prolonging survival [6,7]. Erlotinib, approved by the American Food and Drug Administration (FDA) in 2005 for use with gemcitabine in advanced pancreatic cancer, has demonstrated only minimal efficacy, extending median survival by about two weeks in advanced cases, without significant impacts on disease-free or overall survival in resectable cases [8,9,10]. This limited efficacy is likely due to resistance mechanisms, including secondary mutations and the activation of compensatory signaling pathways [11,12]. Identifying novel agents to potentiate the efficacy of erlotinib remains imperative.

Traditional Chinese medicine (TCM), recognized for its compound diversity, multifaceted target interactions, and generally minimal side effects, is being increasingly studied for its antitumor efficacy [13]. Pien Tze Huang (PZH), a prominent TCM, was found to strengthen intestinal barrier integrity by modulating gut microbiota and metabolites, thus restraining colorectal cancer onset and progression [14]. Additionally, Liujunzi decoction (LJZD) has been shown to bolster T-cell killing ability by downregulating PD-1 expression, leading to the effective suppression of esophageal tumor growth [15].

Network pharmacology integrated with bioinformatics leverages gene and active compound databases to construct a comprehensive “compounds-targets-disease” network, thereby elucidating TCM mechanisms from a holistic systems perspective [16]. The analysis of *Antrodia cinnamomea* through UPLC-Q-TOF/MS identified 139 chemical constituents, with network pharmacology and bioinformatics pinpointing the PI3K/AKT pathway as a likely mechanism for liver cancer growth inhibition, validated through both in vivo and in vitro experiments [17]. The investigation of the Huoxue Yiqi Recipe-2 using network pharmacology suggested its potential anti-lung cancer effects, further supported by in vitro and in vivo data demonstrating its tumor-suppressive activity via PD-L1 downregulation and Akkermansia muciniphila upregulation [18].

Chansu, aka *Venenum Bufonis* (VB), derived from the secretion of the skin and parotoid glands of Bufo gargarizans toads [19], is formulated as a sterile aqueous solution known as Chansu injection (CSI), which has been approved by the China Food and Drug Administration (CFDA). Clinically, CSI is employed to address acute and chronic suppurative infections and is utilized adjunctively in oncology and radiation treatments. Bufadienolides, which represent over 90% of CSI’s composition as determined by HPLC analysis [20], exhibit significant anti-cancer effects across various malignancies, including breast, prostate, and colon cancers [21,22,23]. Nonetheless, the impact and mechanistic action of CSI in pancreatic cancer remain inadequately characterized. This study examined CSI’s antitumor effects on pancreatic cancer cells both in vitro and in vivo, with an additional assessment of its interaction with erlotinib.

## 2. Results

### 2.1. Active Compounds of Chansu Injection

High-performance liquid chromatography (HPLC) analysis identified nine bufadienolides as the primary active compounds in CSI, accounting for over 90% of the 96 μg of bufadienolides per mL in each Chansu injection. The representative fingerprint and compound profile are illustrated in Figure S1. Evidence from prior studies confirmed these bufadienolides as key bioactive components with demonstrated antitumor properties [20,21], highlighting their significance as the principal agents in CSI.

### 2.2. CSI Inhibited the Growth of PDAC Cells by Inducing G2/M Phase Arrest

**Figure 1:**
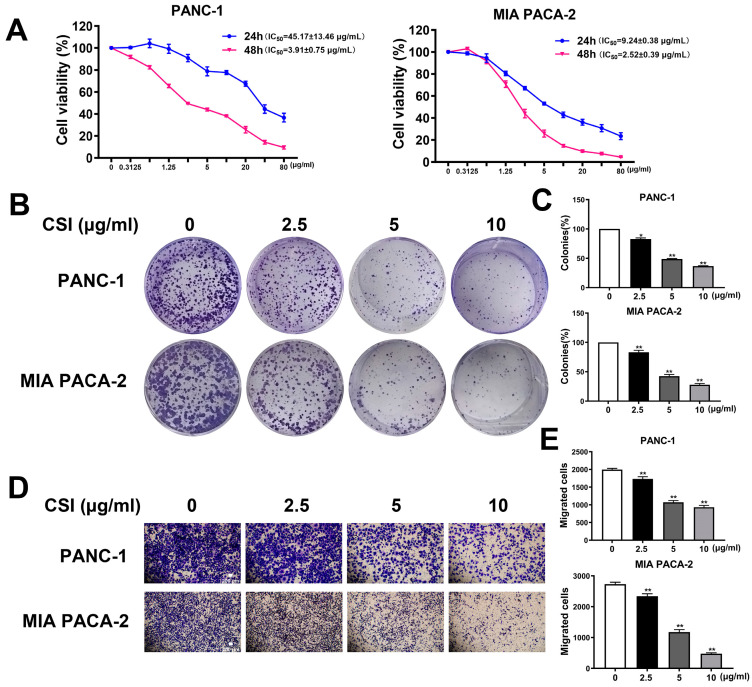
CSI inhibited the proliferation and migration of PDAC cells. (**A**) PANC-1 and MIA PACA-2 cells were treated with 0.1325–80 μg/mL of CSI for 24 h or 48 h, and cell viability was assessed using the MTT assay (n = 3). (**B**,**C**) Colony-formation assays were performed on PANC-1 and MIA PACA-2 cells treated with 2.5, 5 or 10 μg/mL CSI for 48 h (n = 3). (**D**,**E**) Migrating assays were conducted with PANC-1 and MIA PACA-2 cells exposed to 2.5, 5, or 10 μg/mL CSI for 48 h (n = 5). The same volume of sterile water or DMSO was added to the control group. Data were expressed as mean ± standard error (SEM). Statistical significance was defined as * *p* < 0.05 and ** *p* < 0.01 versus the control group.

To assess the inhibitory effects of CSI on the growth of PANC-1 cells (KRAS-G12D mutation) and MIA PACA-2 cells (KRAS-G12C mutation), MTT assays were conducted, yielding average half-maximal inhibitory concentration (IC_50_) values of 45.17 ± 13.46 μg/mL (24 h) and 3.91 ± 0.75 μg/mL (48 h) for PANC-1 cells, and 9.24 ± 0.38 μg/mL (24 h) and 2.52 ± 0.39 μg/mL (48 h) for MIA PACA-2 cells (Figure 1A). Colony-formation assays further demonstrated a reduction in colony numbers (Figure 1B,C), while migration assays showed a decrease in cellular migration (Figure 1D,E), suggesting that CSI effectively impedes both the proliferation and migration of pancreatic cancer cells.

**Figure 2:**
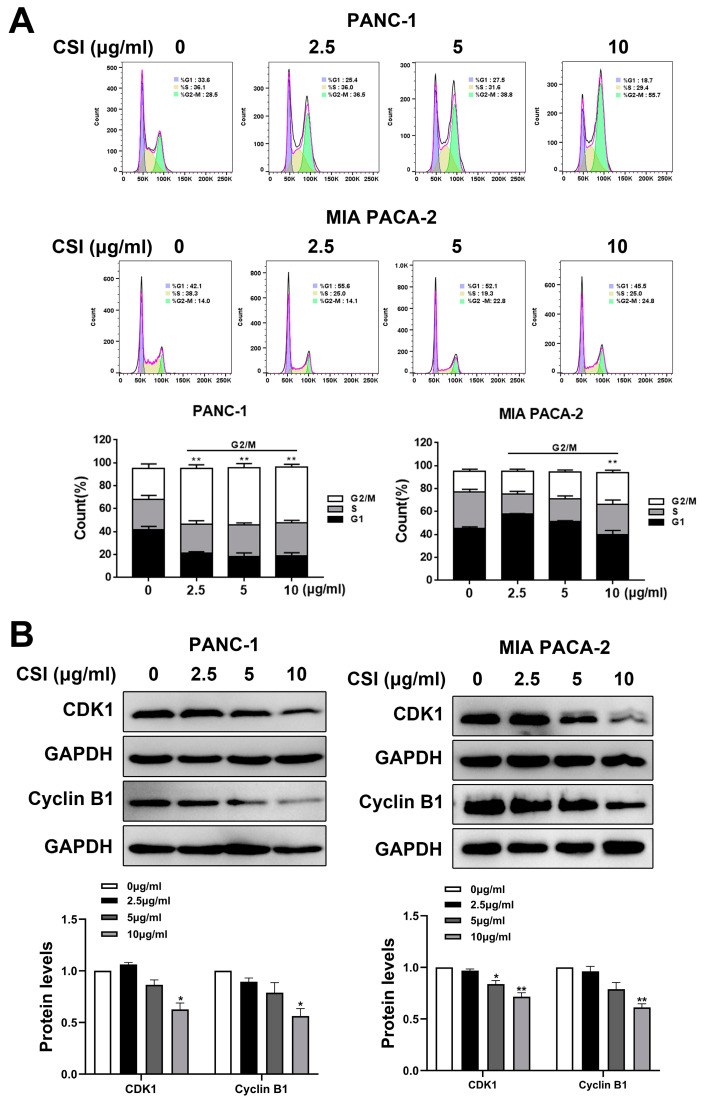
CSI inhibited the growth of PDAC cells by inducing G2/M phase arrest. (**A**) PANC-1 and MIA PACA-2 cells were treated with 2.5, 5, or 10 μg/mL CSI for 24 h, and cell cycle distribution was analyzed using flow cytometry (n = 5). (**B**) The expression of cyclin B1 and CDK1 were determined using Western blot (n = 5). Data were expressed as mean ± standard error (SEM). Statistical significance was defined as * *p* < 0.05 and ** *p* < 0.01 versus the control group.

Flow cytometry analysis indicated that CSI treatment induced G2/M phase arrest in PANC-1 and MIA PACA-2 cells (Figure 2A). Western blot results corroborated this, revealing diminished levels of cyclin-dependent kinase (CDK1) and Cyclin B1 by the treatment of CSI (Figure 2B).

### 2.3. Network Pharmacological and Bioinformatics Analysis of CSI Against PDAC

**Figure 3:**
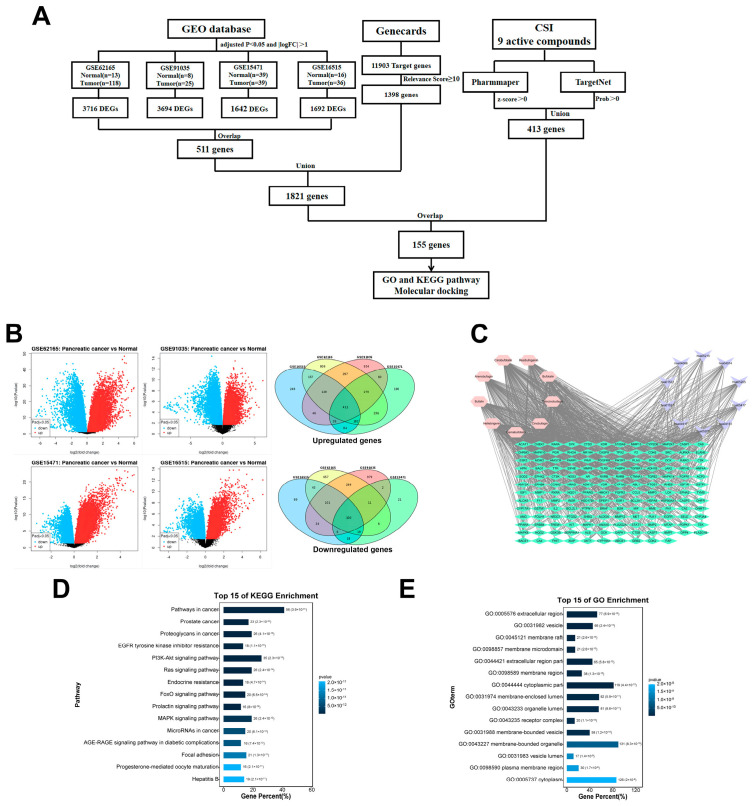
Network pharmacological and bioinformatics analysis of CSI against PDAC. (**A**) Overview of the network pharmacological and bioinformatics analysis for CSI against PDAC. (**B**) Volcano map and Venn diagram depicting differentially expressed genes from GSE62165, GSE91035, GSE15471, and GSE16515. (**C**) PPI network of CSI active compounds and anti-pancreatic cancer targets. (**D**,**E**) GO and KEGG enrichment analyses of 155 common targets for CSI in pancreatic cancer.

**Figure 4:**
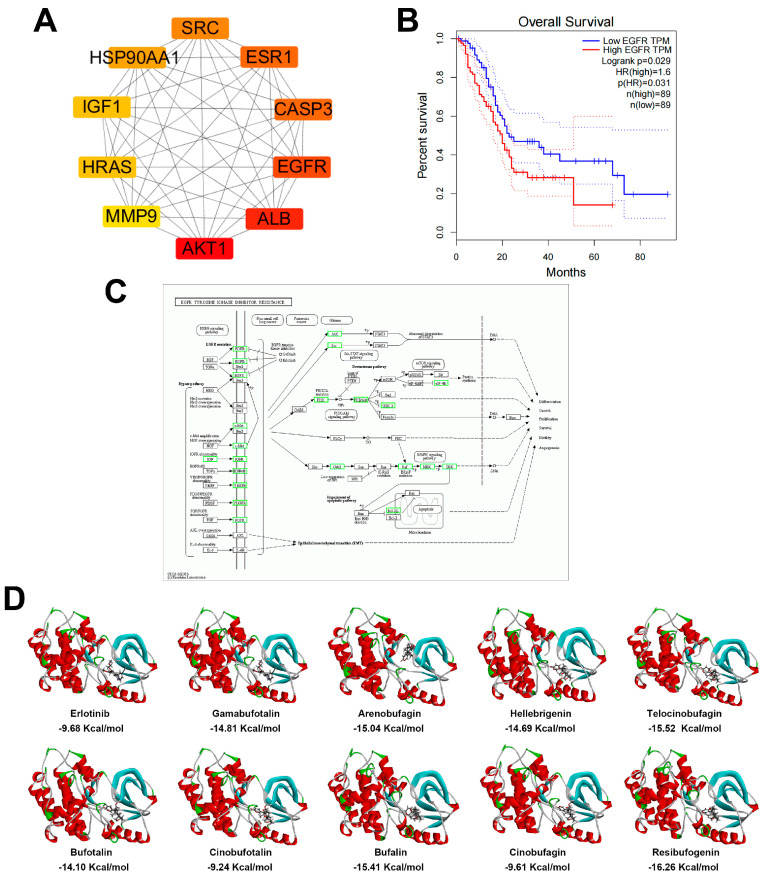
Network pharmacological and bioinformatics analysis of CSI against PDAC. (**A**) Top 10 hub targets of CSI in pancreatic cancer. (**B**) Survival curves of patients with PDAC based on EGFR expression. (**C**) Targets of CSI interactions within the EGFR inhibitor resistance pathway. (**D**) Molecular docking analysis of CSI active compounds with EGFR, with erlotinib used as a positive control.

Network pharmacology and bioinformatics were utilized to identify CSI’s relevant targets and pathways in PDAC treatment (Figure 3A). Gene expression datasets—GSE62165, GSE91035, GSE15471, GSE44077, and GSE16515—were analyzed, comprising 218 pancreatic cancer and 76 normal samples. GEO-2R analysis identified 511 PDAC-associated differentially expressed genes (Figure 3B). Additionally, 1398 pancreatic cancer targets with relevance scores ≥ 10 were extracted from the GeneCards database, yielding 1821 biomarkers when combined. To predict potential targets of CSI’s nine active bufadienolides (Table 1), online tools TargetNet and PharmMapper were applied, revealing an intersection of 155 common targets between CSI and PDAC, potentially underlying CSI’s therapeutic action. A PPI network of these targets was constructed in Cytoscape (Figure 3C), followed by GO and KEGG pathway analyses. KEGG analysis emphasized the role of the EGFR inhibitor resistance pathway in CSI’s effects (Figure 3D and Figure 4C). The PPI network pinpointed AKT1, ALB, and EGFR as core targets (Figure 4A). GEPIA-based survival analysis indicated that among these, only EGFR significantly affects PDAC patient survival (Figure 4B). Molecular docking demonstrated strong affinities of the nine bufadienolides for EGFR, with binding energies lower than −9.24 kcal/mol, including resibufogenin at −16.26 kcal/mol, indicating robust interaction; in comparison, erlotinib’s affinity was −9.68 kcal/mol. These results suggested that CSI’s active components exhibit binding affinities comparable to or exceeding that of erlotinib for EGFR (Figure 4D). Collectively, network pharmacology and bioinformatics analyses implied that CSI’s anti-PDAC actions may operate via EGFR pathway modulation.

### 2.4. CSI Treatment Reduced the Expression of p-EGFR, KRAS, and p-ERK1/2

**Figure 5:**
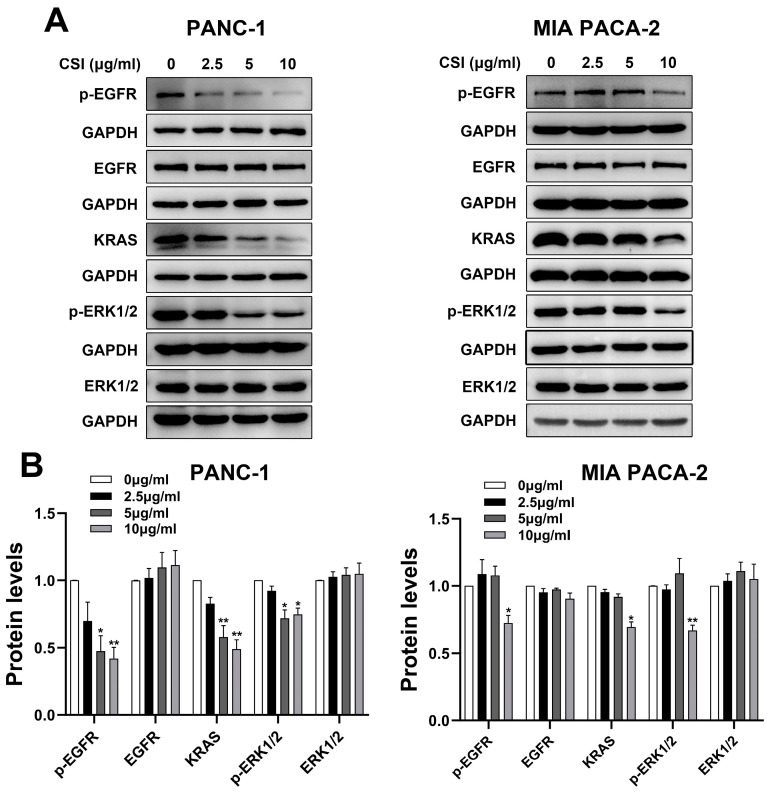
CSI treatment reduced the expression of p-EGFR, KRAS, and p-ERK1/2. (**A**) PANC-1 and MIA PACA-2 cells were treated with 2.5, 5, or 10 μg/mL CSI for 24 h, and the protein expression levels of p-EGFR, EGFR, KRAS, p-ERK1/2, and ERK1/2 were analyzed using Western blot (n = 5). (**B**) Quantification of Western blot results for p-EGFR, EGFR, KRAS, p-ERK1/2, and ERK1/2 (n = 5). Data were expressed as mean ± standard error (SEM). Statistical significance was defined as * *p* < 0.05 and ** *p* < 0.01 versus the control group.

Network pharmacology and bioinformatics analyses indicated that the anti-PDAC effects of CSI were likely mediated via the EGFR signaling pathway. To validate this, expression levels of p-EGFR, KRAS, and p-ERK1/2 were examined using Western blot analysis. The results demonstrated that CSI treatment reduced p-EGFR, KRAS, and p-ERK1/2 levels, while the total EGFR and ERK1/2 levels remained stable (Figure 5A,B), supporting the network pharmacology predictions. Collectively, these data suggest that CSI may exert its anti-PDAC effects through inhibiting the p-EGFR/KRAS/p-ERK1/2 signaling pathway.

### 2.5. Synergistic Effects of CSI and Erlotinib by Inducing G2/M Phase Arrest in PDAC Cells

**Figure 6:**
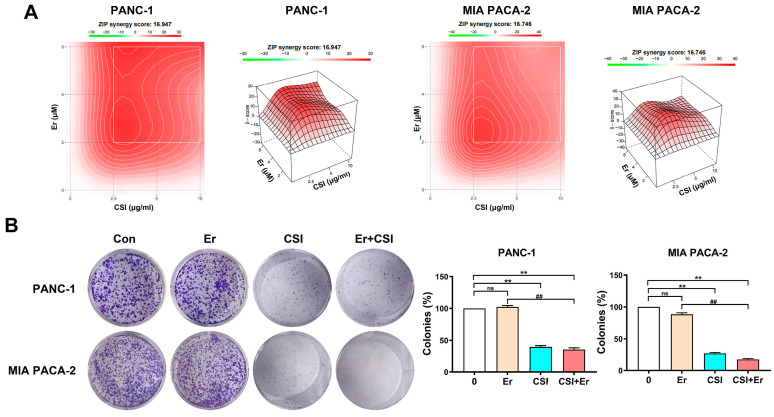
CSI synergistically enhanced the antitumor effect of erlotinib in vitro. Er (erlotinib), CSI (Chansu injection), and Er+CSI (erlotinib+Chansu injection). (**A**) The inhibitory effects of various concentrations of CSI (2.5, 5, 10 μg/mL) in combination with erlotinib (2, 4, 8 μM) on PANC-1 and MIA PACA-2 cells for 48 h were assessed (n = 5). (**B**) Colony-formation assays were conducted on PANC-1 cells and MIA PACA-2 treated with 10 μg/mL CSI, 2 μM erlotinib, or their combination for 48 h (n = 3). Data were expressed as mean ± standard error (SEM). Statistical significance was defined as ** *p* < 0.01 versus the control group, and as ## *p* < 0.01 relative to the erlotinib (Er) group, *ns* represented no significant difference.

Network pharmacology and bioinformatics analyses indicated that CSI’s anti-PDAC effects likely involved the modulation of the EGFR signaling pathway. Molecular docking demonstrated a strong binding affinity between nine bufadienolides and EGFR (Figure 4). The in vivo results further corroborated that CSI exerts anti-PDAC effects via inhibiting the p-EGFR/KRAS/p-ERK1/2 signaling cascade (Figure 5). Although the FDA approved erlotinib in combination with gemcitabine for treating locally advanced, unresectable, or metastatic PDAC, patients frequently develop secondary resistance, limiting erlotinib’s clinical efficacy [24]. Consequently, this study evaluated whether combining CSI with erlotinib could enhance erlotinib sensitivity. PANC-1 and MIA PACA-2 cells were treated with varying concentrations of erlotinib (2, 4, 8 μM) and CSI (2.5, 5, 10 μg/mL) to determine potential synergy. Using SynergyFinder, a drug interaction analysis platform, we determined synergy scores of 16.947 and 16.746 for PANC-1 and MIA PACA-2 cells, respectively. Synergy scores above 10 suggest a synergistic interaction, this indicates a likely synergistic interaction between the CSI and erlotinib (Figure 6A). Colony formation assays further supported this, with combination treatment yielding a greater reduction compared to erlotinib alone (Figure 6B), indicating CSI’s synergistic enhancement of erlotinib cytotoxicity.

**Figure 7:**
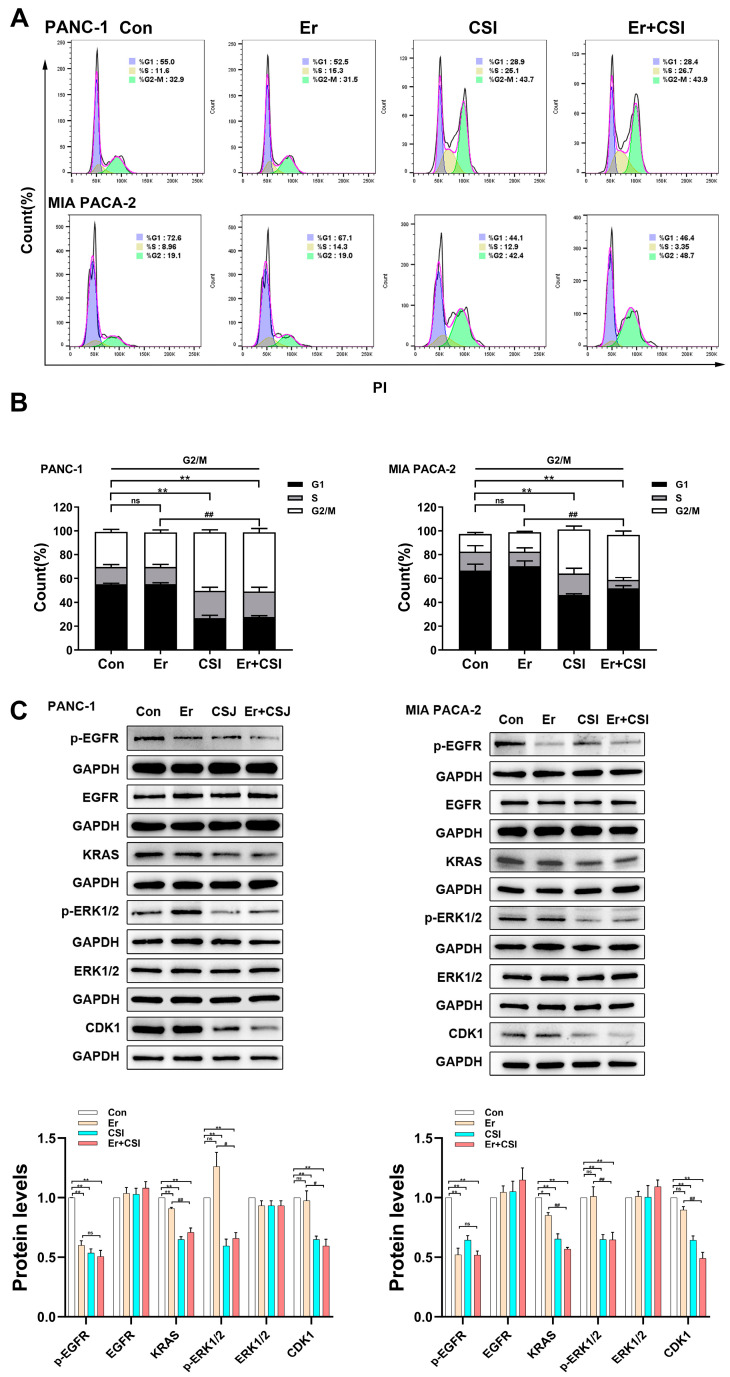
Cotreatment of CSI and erlotinib inhibited KRAS and p-ERK1/2 in PDAC cells. Er (erlotinib), CSI (Chansu injection), and Er+CSI (erlotinib+Chansu injection). (**A**,**B**) PANC-1 and MIA PACA-2 cells were treated with 10 μg/mL CSI, 2 μM erlotinib or their combination for 48 h, and cell cycle distribution was analyzed using flow cytometry assays (n = 5). (**C**) Western blot analysis of p-EGFR, EGFR, KRAS, p-ERK1/2, ERK1/2, and CDK1 (n = 5). Data were expressed as mean ± standard error (SEM). Statistical significance was defined as * *p* < 0.05 and ** *p* < 0.01 versus the control group, as # *p* < 0.05 and ## *p* < 0.01 relative to the erlotinib (Er) group, and *ns* represented no significant difference.

The effects of erlotinib, CSI, and their combination on pancreatic cancer cell cycle dynamics were assessed through flow cytometry (Figure 7A,B). While erlotinib alone showed minimal impact on cell cycle progression, CSI induced G2/M phase arrest, aligning with prior findings (Figure 2). Notably, the combined treatment of CSI and erlotinib further reinforced G2/M phase arrest, accompanied by the downregulation of Cyclin D1 expression in PDAC cells (Figure 7C).

### 2.6. Cotreatment of CSI and Erlotinib Inhibited KRAS and p-ERK1/2 in PDAC Cells

Given that CSI treatment reduced p-EGFR, KRAS, and p-ERK1/2 expression (Figure 5), the subsequent analysis assessed whether combining CSI with erlotinib would produce comparable effects. Western blotting evaluated the p-EGFR/KRAS/p-ERK1/2 signaling pathway in pancreatic cancer cells (PANC-1 and MIA PACA-2) under individual and combined CSI and erlotinib treatments (Figure 7C). Erlotinib alone reduced p-EGFR (Tyr1068) by 40–50% and KRAS by roughly 10%, while p-ERK1/2 (Thr202/Tyr204) remained largely unaffected. In contrast, CSI alone markedly suppressed p-EGFR, KRAS, and p-ERK1/2 levels. The combination therapy notably mitigated erlotinib-induced resistance in KRAS and p-ERK1/2, indicating that CSI potentiates erlotinib’s antitumor efficacy by inhibiting KRAS and p-ERK1/2.

### 2.7. Antitumor Effect of CSI and Erlotinib in Mice

**Figure 8:**
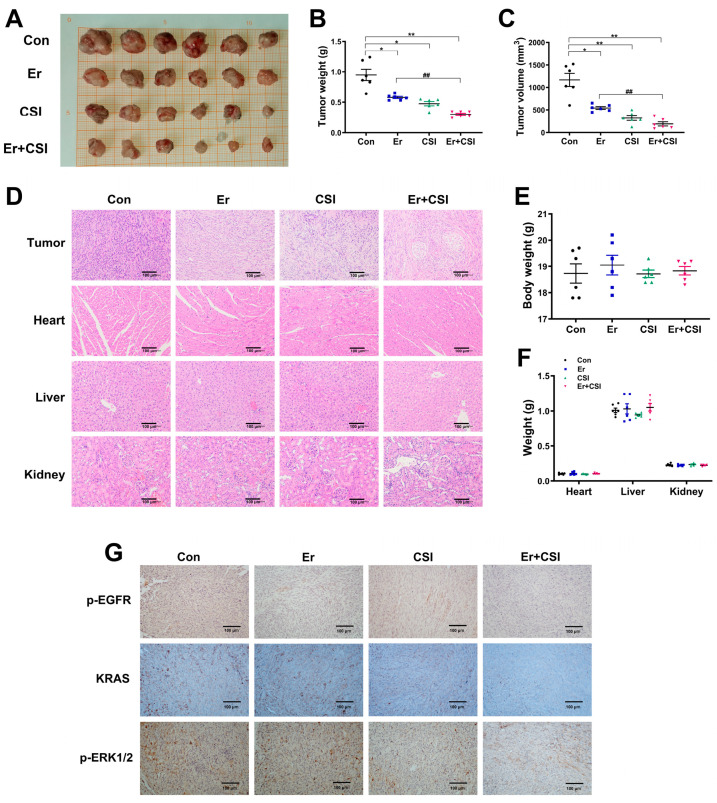
CSI enhanced the antitumor effect of Er (erlotinib) in tumor-bearing mice. (**A**) Images of tumor samples from PDAC-bearing mice (n = 6). (**B**) Effects of CSI and erlotinib combination on tumor weight in tumor-bearing mice (n = 6). (**C**) Effects of CSI and erlotinib combination on tumor volume (n = 6). (**D**) Representative H&E staining images of tumor, heart, liver, and kidney tissues. (**E**) Effect of CSI and erlotinib combination on body weight (n = 6). (**F**) Effect of CSI and erlotinib combination on the weight of the heart, liver, and kidney in mice (n = 6). (**G**) Representative IHC staining images of p-EGFR, KRAS, and p-ERK of tumor. Data were expressed as mean ± standard error (SEM). Statistical significance was defined as * *p* < 0.05 and ** *p* < 0.01 versus the control group, and as ## *p* < 0.01 relative to the erlotinib (Er) group.

In vivo studies examined the therapeutic impact of combining CSI with erlotinib, revealing that both agents independently, as well as their combined use, effectively inhibited tumor growth. Notably, the CSI–erlotinib combination yielded a more substantial reduction in tumor weight and volume compared to monotherapy with either agent (Figure 8A–C). Relative to the erlotinib group, the combination therapy significantly decreased tumor weight and volume in tumor-bearing mice (*p* < 0.01). Hematoxylin–eosin (H&E) staining indicated that treatment with CSI, erlotinib, or their combination induced notable changes in tumor cell morphology, with increased inflammatory cell infiltration and tumor necrosis (Figure 8D). Immunohistochemical (IHC) analysis showed that erlotinib suppressed p-EGFR expression but did not impact KRAS and p-ERK1/2 (Figure 8G). In contrast, CSI treatment reduced p-EGFR, KRAS, and p-ERK1/2 levels. Compared with erlotinib alone, the CSI-erlotinib combination demonstrated enhanced reductions in KRAS and p-ERK1/2. Furthermore, body, heart, liver, and kidney weights showed no significant difference from controls (Figure 8E,F), and H&E staining of these organs confirmed the absence of overt toxicity across treatment groups (Figure 8D–F). The findings indicated that CSI may augment the antitumor efficacy of erlotinib in tumor-bearing mice by inhibiting KRAS and p-ERK1/2, highlighting the potential of this combination therapy for PDAC management.

## 3. Discussion

CSI, a complex extract derived from Chansu, comprises nine key bufadienolide compounds: gamabufotalin, arenobufagin, resibufogenin, hellebrigenin, telocinobufagin, bufalin, bufotalin, cinobufagin, and telocinobufagin. Existing studies predominantly explore the individual anti-cancer effects of these bufadienolides. Gamabufotalin, for example, effectively inhibits tumor cell proliferation in osteosarcoma, hepatoma, and glioblastoma [25,26,27] and enhances temozolomide efficacy in glioblastoma [28]. Hellebrigenin and arenobufagin induce G2/M phase arrest, reducing the growth of ER-positive breast cancer and glioblastoma cells [29,30]. Telocinobufagin blocks STAT3 signaling in non-small-cell lung cancer and osteosarcoma [31,32], while bufotalin induces ferroptosis by GPX4 degradation, thus inhibiting non-small-cell lung cancer progression [33]. Cinobufotalin shows efficacy against colon adenocarcinoma and diminishes cisplatin resistance in lung adenocarcinoma and hepatocellular carcinoma via c-Myc degradation through ubiquitination [34,35,36]. Bufalin exhibits notable anti-cancer and immune-modulatory activities, promoting M2-to-M1 macrophage polarization in hepatocellular and colorectal cancers [37,38,39,40,41,42,43,44]. Cinobufagin also restricts nasopharyngeal carcinoma metastasis by reducing UBE3A-mediated p53 ubiquitination [45]. Resibufogenin inhibits TAK1-mediated NF-κB signaling in PDAC through a PKC-dependent pathway [46]. Clinically, CSI is applied as an adjuvant cancer therapy not a single compound, with our findings demonstrating that CSI-derived bufadienolides notably suppress pancreatic cancer cell proliferation by inducing G2/M arrest, potentially via the p-EGFR/KRAS/p-ERK1/2 pathway (Figure 5).

A defining characteristic of PDAC is its abundant stroma, comprising fibroblasts, immune cells, inflammatory cells, growth factors, and an extensive extracellular matrix (ECM) [47]. Chemotherapeutic agents must penetrate this ECM to reach the cancer cell cytoplasm and exert cytotoxic effects. Fibroblasts constitute nearly 90% of the pancreatic tumor volume, primarily consisting of a heterogeneous subset of cancer-associated fibroblasts (CAFs), among which myofibroblastic CAFs (myCAFs) serve as the predominant cellular component of the PDAC stroma [48]. Activated by pancreatic cancer cells, CAFs proliferate and produce ECM proteins, creating a physical barrier that obstructs immune cell infiltration and hinders drug delivery, thereby fostering resistance to chemotherapy and immunotherapy. Inflammatory CAF activation within PDAC is predominantly driven by interleukin-1 (IL-1) and transforming growth factor β (TGF-β) signaling pathways [49]. The treatment of pancreatic stellate cells—the progenitors of CAFs in PDAC—with TGF-β results in the marked upregulation of EGFR and HER2 activation, significantly enhancing the pro-metastatic capacity of myCAFs and promoting cancer cell metastasis in PDAC [50]. Additionally, KRAS mutations in pancreatic cancer induce fibroblast autocrine signaling, elevating cytokine interleukin-33 (IL-33) secretion within the stroma. This stromal IL-33 decreases CD8^+^ T-cell infiltration and activation, consequently facilitating tumor progression [51].

EGFR, which binds to the epidermal growth factor, initiates downstream signaling cascades that drive cell division and proliferation. Overexpressed in 57–95% of pancreatic cancer patients, EGFR activation frequently occurs concurrently with cancer progression and metastasis [52]. As an upstream regulator of RAS, EGFR is integral to KRAS-driven cancers [52]. The RAS protein family—KRAS, NRAS, and HRAS—consists of small GTPases, with KRAS mutations detected in 86–91% of pancreatic cancer cases [53]. Under normal conditions, KRAS cycles between active and inactive states; however, mutations result in constitutive activation, fueling uncontrolled tumor growth [54]. In pancreatic ductal adenocarcinoma (PDAC), the most prevalent KRAS mutations are found at codon 12, with G12D (45%), G12V (35%), and G12R (17%) as dominant forms, alongside less common G12C and G12F mutations [55]. Historically challenging to target, KRAS mutations have seen breakthroughs with FDA-approved inhibitors such as Sotorasib (AMG510) and Adagrasib (MRTX849) for G12C variants [56,57]. A novel agent, Divarasib (GDC-6036), demonstrates selective inhibition against KRAS-G12C, effectively locking the mutated KRAS in an inactive state to block oncogenic signaling [58]. Preclinical studies reveal Divarasib’s efficacy to be 5–20 times higher than Sotorasib and Adagrasib, with a 50-fold increase in selectivity [59]. In a phase 1 trial on advanced or metastatic tumors with KRAS G12C mutations, Divarasib achieved an objective response rate (ORR) of 56.4% in non-small-cell lung cancer patients and a median progression-free survival (PFS) of 13.1 months. For colorectal cancer patients, ORR and PFS were 35.9% and 6.9 months, respectively [58]. Clinical Trial NCT04449874 demonstrated that the ORR of Divarasib combined with cetuximab (EGFR monoclonal antibody) in colon cancer patients with KRAS G12C mutations reached 62.5%, surpassing the ORRs of other KRAS G12C inhibitors, including sotorasib+panitumumab (26.4%) and adagrasib+cetuximab (46%) reported in previous studies [60,61,62]. Furthermore, the PFS of Divarasib with cetuximab extended to 8.1 months, representing the longest duration among all KRAS G12C inhibitor clinical trials for metastatic colorectal cancer. Additionally, treatment-related adverse events in all patients were predominantly mild, with 91.6% categorized as grade 1–2 [62]. However, in pancreatic cancer, G12D and G12V mutations are predominant, representing approximately 80% of cases, which limits the applicability of G12C-targeted therapies in this patient population.

KRAS mutations diminish the efficacy of EGFR-targeted therapies. In metastatic colorectal cancer patients harboring KRAS mutations (KRAS G12C), treatment with EGFR inhibitors or monoclonal antibodies yields minimal benefit [63,64]. For KRAS(G12C) inhibitors, MAPK signaling pathway reactivation serves as a primary mechanism of resistance, predominantly mediated by EGFR, which reinitiates RAS-MAPK pathway signaling and constrains therapeutic impact [65,66]. Therefore, the adaptive feedback reactivation of the EGFR/RAS/MAPK signaling pathway contributes to resistance against KRAS inhibitors. Furthermore, evidence supports the enhanced efficacy of combining KRAS-targeted treatments with EGFR inhibition over monotherapy approaches [67,68]. KRAS mutations reduce the efficacy of erlotinib, as evidenced in patients with non-small-cell lung cancer where KRAS mutations led to diminished progression-free survival with erlotinib plus chemotherapy [69]. In cell line studies, erlotinib treatment reduced p-MAPK levels in BxPC-3 cells (KRAS wild type), while MIA PaCa-2 cells (KRAS-G12C mutation) exhibited minimal response [70]. Likewise, PANC-1 (KRAS-G12D mutation) and MIA PaCa-2 cells (KRAS-G12C mutation) demonstrated erlotinib resistance, as indicated by persistent p-AKT and p-ERK1/2 levels [71]. These results are consistent with our findings, underscoring the role of sustained KRAS activation in mediating PDAC resistance. In the present study, the combination of CSI with erlotinib effectively inhibited erlotinib-induced resistance pathways involving KRAS and p-ERK1/2 (Figure 7). Additionally, CSI showed no notable toxicity to the kidneys, heart, or liver in mice at therapeutic doses, suggesting its safety profile. The synergistic effect of CSI and erlotinib thus offers a promising approach to counteract KRAS mutation-associated resistance in PDAC. Limitations of this study should be noted. Given that Chansu is a multi-component formulation, further investigation into its metabolism and distribution in vivo will be conducted using metabolomics, alongside RNA-sequencing or proteomics to elucidate the mechanisms of its active compounds. Future studies will also explore Chansu’s potential to inhibit PDAC proliferation by examining various aspects, including the tumor immune microenvironment and inflammatory responses.

## 4. Materials and Methods

### 4.1. Chemicals and Reagents

Chansu injection was sourced from Jiangsu Pujin Pharmaceutical Co., Ltd. (Nanjing, China. approval number: No. Z32020694; batch number: 20210706). Erlotinib (10 μM stock solution in DMSO) was supplied by MedChemExpress Bio-Tech Co., Ltd (Shanghai, China). MTT and RIPA lysis buffers were obtained from Beyotime (Shanghai, China). Antibodies targeting p-EGFR (Tyr1068), EGFR, p-ERK1/2 (Thr202/Tyr204), and ERK1/2 were procured from Cell Signalling Technology (Danvers, MA, USA). GAPDH and HRP-conjugated goat anti-rabbit (mouse) IgG were provided by Proteintech (Wuhan, China), while antibodies against BCL-2, BAX, CDK1, and Cyclin B1 were acquired from Boster (Wuhan, China).

### 4.2. Active Compounds of Chansu Injection by High-Performance Liquid Chromatography (HPLC) Anaysis

Chansu injection compounds were analyzed using an Agilent 1200 high-performance liquid chromatography system equipped with an Agilent Zorbax SB C-18 column (250 mm length, 4.6 mm internal diameter, 5 μm particle size). The mobile phase consisted of acetonitrile and 0.1% ammonium acetate, set to a flow rate of 1.0 mL/min at a column temperature of 30 °C, with detection at a UV wavelength of 296 nm. The theoretical plate count, calculated based on the bufalin peak, exceeded 3000. Precisely measured quantities of bufalin, resibufogenin, and cinobufagin were diluted in a 50% acetonitrile solution to achieve a standard concentration of 1.6 μg/mL (each 1 mL contains 1.6 μg of bufalin, resibufogenin, and cinobufagin). Following this, 50 μL each of the standard solution and Chansu injection were accurately injected into the HPLC. The external standard method was employed to quantify bufalin, resibufogenin, and cinobufagin in Chansu injection, ensuring a concentration range of 1.0–5.0 μg per mL.

### 4.3. Cell Lines and Cell Culture

PANC-1 and MIA PACA-2 cell lines were sourced from the Cell Bank of the Chinese Academy of Sciences (Shanghai, China). PANC-1 cells were maintained in DMEM (Gibco, Grand Island, NY, USA) with 10% fetal bovine serum and 1% penicillin–streptomycin, while MIA PACA-2 cells were cultured in DMEM supplemented with 10% fetal bovine serum, 2.5% horse serum, and 1% penicillin–streptomycin. Incubation conditions for all cultures were set at 37 °C with 5% CO_2_ and saturated humidity.

### 4.4. MTT Assay

Cell viability was assessed using the MTT assay. PANC-1 and MIA PaCa-2 cells (6 × 10^3^ cells per well) were plated in 96-well plates and allowed to adhere for 24 h before treatment with varying concentrations of CSI or erlotinib, while control wells received an equivalent volume of DMSO (<0.1%) or sterile water. Following a 4 h incubation with the MTT solution (5 mg/mL), the supernatant was carefully removed, and 150 µL of DMSO was added to dissolve the formazan crystals. Absorbance at 490 nm was then recorded with a microplate reader.

### 4.5. Colony-Formation Assay

Pancreatic cancer cells (2000 cells/well) were seeded in 6-well plates and allowed to adhere overnight. Following attachment, cells were exposed to CSI or erlotinib for 48 h, after which the medium was refreshed every two days, continuing the culture for an additional week. Colonies were subsequently stained with crystal violet.

### 4.6. Cell Cycle Analysis

Cell cycle distribution was evaluated following a standard protocol. A total of 1 × 10^6^ cells per well were seeded in 6-well plates and incubated overnight. After 24 h treatments with CSI or erlotinib, cells were harvested using 0.25% trypsin and fixed in 70% ethanol at −20 °C overnight. Following two PBS washes, cells were stained with propidium iodide (50 μg/mL) and RNase A (100 μg/mL) for 30 min in darkness. A BD Accuri C6 flow cytometer (San Jose, CA, USA) was used for analysis, and FlowJo V10 software (TreeStar, Woodburn, OR, USA) quantified cell cycle distribution ratios.

### 4.7. Western Blot Analysis

Following CSI or erlotinib treatment, cells were washed twice with cold PBS, lysed in an ice-cold RIPA buffer containing protease and phosphatase inhibitors, and subsequently sonicated and centrifuged. Protein concentrations were determined via the BCA assay, after which equal protein quantities (30–50 μg) were resolved by SDS-PAGE and transferred onto PVDF membranes (Millipore, Bedford, MA, USA). To minimize non-specific binding, 5% nonfat milk or BSA was applied before overnight incubation with primary antibodies at specified dilutions. Post wash, membranes were incubated with corresponding secondary antibodies for 2 h. Enhanced chemiluminescent substrate (Biosharp Laboratories Co., Ltd., Hefei, China) was used to visualize immunoreactivity, and band intensities were quantified using Image-J 1.53q software (Rawak Software Inc., Stuttgart, Germany), with values normalized to respective control groups.

### 4.8. Animal Treatment with CSI

Female C57BL/6 mice (4–6 weeks old) were subcutaneously injected with a PAN02 cell suspension (1 × 10^7^ cells/mouse). Once tumors reached approximately 100 mm^3^ (about 10 days), mice were randomly allocated to four groups: Control (0.1 mL/10 g solvent), Erlotinib (10 mg/kg), CSI (0.1 mL/10 g), and Erlotinib–CSI combination (10 mg/kg Erlotinib + 0.1 mL/10 g CSI). Erlotinib was dissolved in 0.5% CMC-Na/saline. All treatments were delivered by daily intraperitoneal injection over a 14-day period. Tumor volume was calculated using the formula: volume (mm^3^) = 0.5 × length (mm) × [width (mm)]^2^. Following treatment, mice were euthanized, and tumors and other organs were excised and weighed. All procedures complied with Shihezi University’s Animal Care and Use Policy (Ethical Code: A2024-033-01).

### 4.9. HE Staining

Tumor tissue was fixed in 4% paraformaldehyde for 24 h, dehydrated in ethanol until transparent, then embedded in paraffin, sectioned, dewaxed, and rehydrated. Sections were stained with hematoxylin for 5 min, decolorized with 1% hydrochloric acid ethanol, and subsequently stained with eosin for 3 min, followed by rinsing in running water. After dehydration, sections were sealed with neutral resin and observed under a microscope, with images captured for analysis.

### 4.10. Immunohistochemistry (IHC)

Tissue sections were dewaxed, hydrated, and subjected to microwave antigen retrieval using 0.1 M sodium citrate solution. To block endogenous peroxidase activity, sections were treated with 3% H_2_O_2_ for 20 min. Following a rinse, sheep serum was applied to minimize non-specific staining. Primary antibodies against p-EGFR (1:200), KRAS (1:100), and p-ERK1/2 (1:400) were then incubated overnight at 4 °C in a humidified chamber. After washing, sections were treated with a secondary antibody at 37 °C for 30 min. Subsequent washes were followed by staining with diaminobenzidine (DAB) solution, counterstaining with hematoxylin, dehydration, and sealing with neutral gum for microscopic examination.

### 4.11. Enrichment Analysis of Common Target Between CSI and Pancreatic Cancer by Network Pharmacology and Bioinformatics

Gene expression profiles for pancreatic cancer (GSE62165, GSE91035, GSE15471, and GSE16515) were retrieved from the GEO database (https://portal.gdc.cancer.gov/, accessed on 9 July 2021). GEO-2R analysis identified differentially expressed genes with significance thresholds of *p* < 0.05 and |log2FC|>1. Validated pancreatic cancer targets from GeneCards (http://www.genecards.org/, accessed on 9 July 2021) were integrated with predicted targets to establish pancreatic cancer biomarkers. The target prediction for the nine active CSI compounds was conducted via TargetNet (http://targetnet.scbdd.com/home/index/, accessed on 18 June 2021) and PharmMapper (http://www.lilab-ecust.cn/pharmmapper/, accessed on 21 June 2021), and the overlapping of these targets with PDAC biomarkers identified potential anti-pancreatic cancer targets of CSI. The protein–protein interaction (PPI) network was constructed using String (https://string-db.org, accessed on 4 November 2021) and Cytoscape 3.6.0. GO and KEGG enrichment analyses were performed with the DAVID bioinformatic database (https://david.ncifcrf.gov/, accessed on 28 August 2022). Survival analysis for AKT1, ALB, and EGFR was conducted through GEPIA (http://gepia.cancer-pku.cn/index.html, accessed on November 15, 2022). Molecular docking utilized AutoDock4 (version 4.2.6), and the EGFR–ligand interactions were visualized using Discovery Studio™ 2.5 (DS; Accelrys Software Inc., San Diego, CA, USA).

### 4.12. Statistical Analysis

Data were expressed as mean ± standard error (SEM) and were analyzed with GraphPad 8.0 software (San Diego, CA, USA). Statistical analyses for multiple group comparisons were conducted using one-way ANOVA in SPSS 23.0 (IBM, Chicago, IL, USA). Statistical significance was defined as * *p* < 0.05 and ** *p* < 0.01 versus the control group, and as # *p* < 0.05 and ## *p* < 0.01 relative to the erlotinib (Er) group.

## 5. Conclusions

KRAS mutations reduce the clinical efficacy of erlotinib in pancreatic cancer treatment. As an adjuvant used in antitumor and anti-radiation therapies, CSI enhances erlotinib’s antitumor effects through inhibiting of the KRAS pathway, suggesting CSI’s potential as an adjunctive therapy specifically targeting KRAS-mutated pancreatic cancer. The dual targeting of KRAS mutations and EGFR demonstrated greater therapeutic effectiveness in pancreatic cancer management compared to single-agent approaches.

## Figures and Tables

**Table 1:** The active compounds of CSI.

No.	Compounds	Chemical Formula	Molecular Weight
1	Gamabufotalin	C_24_H_34_O_5_	402.5
2	Arenobufagin	C_24_H_32_O_6_	416.5
3	Hellebrigenin	C_24_H_32_O_6_	416.5
4	Telocinobufagin	C_24_H_34_O_5_	402.5
5	Bufotalin	C_26_H_36_O_6_	444.6
6	Cinobufotalin	C_26_H_34_O_7_	458.5
7	Bufalin	C_24_H_34_O_4_	386.5
8	Cinobufagin	C_26_H_34_O_6_	442.5
9	Resibufogenin	C_24_H_32_O_4_	384.5

## Data Availability

The raw data supporting the conclusions of this article will be made available by the authors upon request.

## Supplementary Materials

The following supporting information can be downloaded at: https://www.mdpi.com/article/10.3390/ph17121696/s1, Figure S1: Active compounds of Chansu injection by HPLC anaysis. 5-hydroxytryptamine (1), bufotenine (2), gamabufotalin (3), arenobufagin (4), hellebrigenin (5), telocinobufagin (6), bufotalin (7), cinobufotalin (8), bufalin (9), cinobufagin (10), resibufogenin (11).
