# Supplementary material for: Bufadienolides from Chansu Injection Synergistically Enhances the Antitumor Effect of Erlotinib by Inhibiting the KRAS Pathway in Pancreatic Cancer
Source: Pharmaceuticals (Basel). 2024 Dec 16;17(12):1696. doi: 10.3390/ph17121696 (PMC11677899; doi:10.3390/ph17121696)
Supplement: Supplementary file 1 [file pharmaceuticals-17-01696-s001.zip › pharmaceuticals-3244089-supplementary.pdf]

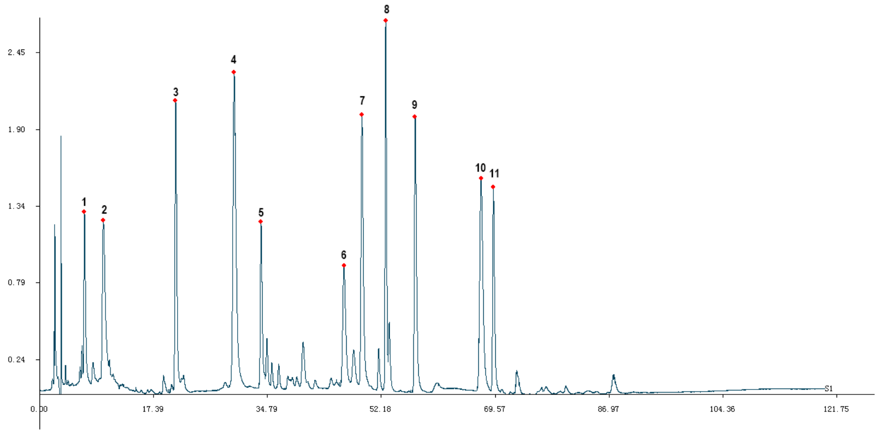

Figure S1. Active compounds of Chansu injection by HPLC anaysis. 5-hydroxytryptamine (1), bufotenine (2), gamabufotalin (3), arenobufagin (4), hellebrigenin (5), telocinobufagin (6), bufotalin (7), cinobufotalin (8), bufalin (9), cinobufagin (10), resibufogenin (11).
